# Effectiveness of Short-Term Heat Acclimation on Intermittent Sprint Performance With Moderately Trained Females Controlling for Menstrual Cycle Phase

**DOI:** 10.3389/fphys.2019.01458

**Published:** 2019-11-29

**Authors:** Andrew T. Garrett, Edward Dodd, Victoria Biddlecombe, Damien Gleadall-Siddall, Rachel Burke, Jake Shaw, James Bray, Huw Jones, Grant Abt, Jarrod Gritt

**Affiliations:** ^1^Department of Sport, Health and Exercise Science, Faculty of Health Science, University of Hull, Hull, United Kingdom; ^2^Mathematics and Physical Science, Faculty of Science and Engineering, University of Hull, Hull, United Kingdom

**Keywords:** female, menstrual cycle, dehydration, fluid-regulation, plasma volume

## Abstract

**Introduction:**

Investigate the effectiveness of short-term heat acclimation (STHA), over 5-days (permissive dehydration), on an intermittent sprint exercise protocol (HST) with females. Controlling for menstrual cycle phase.

**Materials and Methods:**

Ten, moderately trained, females (Mean [SD]; age 22.6 [2.7] y; stature 165.3 [6.2] cm; body mass 61.5 [8.7] kg; $\dot{\text{V}}\,{\text{O}}_{2\,\mathrm{peak}}$ 43.9 [8.6] mL⋅kg^–1^⋅min^–1^) participated. The HST (31.0°C; 50%RH) was 9 × 5 min (45-min) of intermittent exercise, based on exercise intensities of female soccer players, using a motorized treadmill and Wattbike. Participants completed HST1 vs. HST2 as a control (C) trial. Followed by 90 min, STHA (no fluid intake), for five consecutive days in 39.5°C; 60%RH, using controlled-hyperthermia (∼rectal temperature [T_re_] 38.5°C). The HST3 occurred within 1 week after STHA. The HST2 vs HST3 trials were in the luteal phase, using self-reported menstrual questionnaire and plasma 17β-estradiol.

**Results:**

Pre (HST2) vs post (HST3) STHA there was a reduction at 45-min in *T*_*re*_ by 0.20°C (95%CI −0.30 to −0.10°C; *d* = 0.77); ${\bar{T}}_{s\,k}$ (−0.50; −0.90 to −0.10°C; *d* = 0.80); and ${\bar{T}}_{b}$ (−0.25; −0.35 to −0.15°C; *d* = 0.92). Cardiac frequency reduced at 45-min (−8; −16 to −1 b⋅min^–1^; *d* = 1.11) and %PV increased (7.0; −0.4 to 14.5%: *d* = 1.27). Mean power output increased across all nine maximal sprints by 56W (−26 to 139W; *d* = 0.69; n = 9). There was limited difference (*P* > 0.05) for these measures in HST1 vs HST2 C trial.

**Discussion:**

Short-term heat acclimation (5-days) using controlled-hyperthermia, leads to physiological adaptation during intermittent exercise in the heat, in moderately trained females when controlling for menstrual cycle phase.

## Introduction

The worldwide popularity of football results in competitive matches being held in a whole host of environmental conditions, some of which can be in excess of 30°C with high levels of RH (Ozgunen et al., 2010). The 2016 Olympic Games in Brazil and the future 2020 Olympics in Tokyo, Japan (Gerrett et al., 2019) are examples of this.

It has been reported that the menstrual cycle plays a significant role in athletic performance in the heat (Avellini et al., 1979; Tenaglia et al., 1999; Janse de Jonge et al., 2012). However, research is limited and recent evidence has been contradictory. It has demonstrated little effect of the menstrual cycle (Sunderland and Nevill, 2003), oral contraceptive pill (OCP) usage (Lei et al., 2017) and in trained females who have smaller fluctuations in hormonal response (Lei and Mundel, 2018). However, the possible attenuation of endurance performance during heat stress has been reported (Avellini et al., 1979; Tenaglia et al., 1999; Constantini et al., 2005; Janse de Jonge et al., 2012) but this is not a universal finding (Kolka and Stephenson, 1997; Sunderland and Nevill, 2003; Lei et al., 2017). Secondly, there is limited research on fluid handling across the menstrual cycle with OCP usage (Stachenfeld et al., 1999; Stachenfeld, 2008). This supports the need for a robust research design in the present study. It is recognized that resting core temperature and the temperature threshold for the activation of the thermoregulatory responses, cutaneous vasodilation and sweating in females is elevated during the luteal phase of the menstrual cycle (Stachenfeld et al., 2000; Sunderland and Nevill, 2003). Especially, in those taking the OCP (Charkoudian and Johnson, 1997; Rogers and Baker, 1997) which can rise by ∼0.2–0.6°C in the early follicular phase (Stephenson and Kolka, 1985; Kolka and Stephenson, 1989; Grucza et al., 1993; Rogers and Baker, 1997). Many women feel that the menstrual cycle status negatively affects athletic performance (Wilson et al., 1991; Bruinvels et al., 2016) but in reality it has been reported that most physical aspects of athletic performance are not hindered by the menstrual cycle (Lebrun et al., 1995; Constantini et al., 2005; Wiecek et al., 2016; Tounsi et al., 2017). In summary, research on how the menstrual cycle phase affects exercise performance in unacclimated females in the heat is limited and contradictory. Furthermore, the limited information on female sex hormones, OCP and fluid regulation by Stachenfeld and colleagues (Stachenfeld et al., 1999; Stachenfeld, 2008) supports the research design of the present study.

There are fundamentally three models in which active heat acclimation can be achieved; (i) constant work-rate, (ii) self-regulated exercise and (iii) the controlled hyperthermia or isothermic technique (Taylor and Cotter, 2006). The controlled hyperthermia technique has been postulated to provide greater heat adaptation than the constant work-rate and self-regulated work-rate methodologies. On the basis that core temperature elevation is a key consideration for successful heat acclimation associated with high skin temperature and sweating response, as exercise alone is not a sufficient stimulus for adaptation (Hessemer et al., 1986). In contrast, there has been evidence to demonstrate isothermic and fixed intensity heat acclimation methods induce similar heat adaptation in the short and long-term. However, it is suggested controlled-hyperthermia is a more efficient and practical method for heat adaptation, especially for athletes tapering before competition (Gibson et al., 2015). The addition of a permissive dehydration stimulus, that is restricting fluid intake during acclimation has received recent attention in the literature (Akerman et al., 2016) and with a female cohort (Kirby et al., 2019). However, the benefits of permissive dehydration has not been reported universally (Neal et al., 2016). In our previous work using a male cohort (Garrett et al., 2014), permissive dehydration during acclimation has been shown to improve the fluid regulatory mechanisms by improving the reabsorption of water and *Na*^+^ resulting in PV expansion. This indicated that the adaptive response may be enhanced rather than impaired by dehydration acclimation and this work had a euhydration control. Furthermore, we have demonstrated that the use of permissive dehydration with STHA (5-days) has been shown to provide heat adaptation for moderately (Garrett et al., 2009) and highly trained (Garrett et al., 2012) male athletes. From a practical perspective it provides a very light exercise load that minimizes both additional exercise strain and the disruption of quality training during the tapering period for competition (Garrett et al., 2011).

Most heat acclimation protocols have been carried out using male cohorts. Furthermore, the limited research that has been conducted using female participants, to the author’s knowledge only three have used the controlled hyperthermia technique. Mee et al. (2015) reported that in response to STHA of 5-days with permissive dehydration, there was limited physiological change. Improvements in cardiovascular stability, lower core temperature and sudomotor adaptation required a longer, medium-term heat acclimation (MTHA) of 10-days (Mee et al., 2015). Similarly, Kirby et al. (2019) determined nine-, but not 4-days heat acclimation with permissive dehydration, improves self-paced endurance performance in females (Kirby et al., 2019). Daanen and Herweijer (2015) demonstrated limited responses to STHA of 3-days in a younger versus an older female population (Daanen and Herweijer, 2015). However, these studies may not give us all the information we need given their research designs have mixed OCP and non-users. Furthermore, they have not controlled for menstrual cycle phase.

The aims of the present study are to evaluate the physiological and performance effects of a STHA (5-day) protocol, using the controlled hyperthermia technique with no fluid intake, on an intermittent HST with a female cohort and controlling for menstrual cycle phase. It is hypothesized that STHA will improve thermoregulation in a cohort of young, moderately trained female game players when controlling for menstrual cycle phase.

## Materials and Methods

### Experimental Design and Overview

**FIGURE 1:**
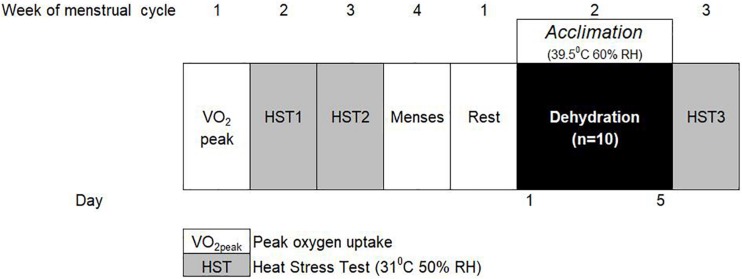
Schematic model of the short-term heat acclimation (STHA) protocol for moderately trained females.

Ten moderately trained female participants undertook a 5-day STHA regime, with no fluid replenishment during each daily acclimation session. Participants’ thermoregulatory, cardiovascular and fluid-regulatory status were measured at rest and in response to an intermittent, exercising HST, administered the week before and after the 2nd day after the STHA regime to ensure 1 day of rest. Participants were asked to refrain from strenuous exercise immediately before and 24 h prior to each HST, as it has been demonstrated that lower resting core temperature contributes to reduced physiological strain during acclimation (Kampmann et al., 2008). A general overview of the STHA protocol for the moderately trained females is shown in Figure 1.

### Participants

Ten, moderately trained, females (Mean [SD]; age 22.6 [2.7] years; stature 165.3 [6.2] cm; body mass 61.5 [8.7] kg; cardiac output 5.5 [1.3] L; and $\dot{\text{V}}\,{\text{O}}_{2\,\mathrm{peak}}$ 43.9 [8.6] mL⋅kg^–1^⋅min^–1^) participated. They were games players and oral contraceptive pill users (combined). Participants completed pre-exercise medical questionnaires and informed consent to participate in the study. All participants were in good health. The study had ethical approval (No. 1516177) from the University of Hull ethics committee following the World Health Organization declaration of Helsinki guidelines.

### Protocol

#### Experimental Standardization

All participants were fully informed of all experimental procedures (orally and written). Prior to experimental testing participants completed pre-exercise medical questionnaires and informed consents. Each female participant used a monophasic, oral contraceptive pill (OCP) and dose of hormone concentration differed between individuals depending on their specific medication. All participants were previously unacclimated to the heat and this study was completed outside the British summertime to minimize seasonal acclimatization effects. To minimize circadian rhythm affects HSTs and acclimations occurred at the same time of day. Participants were asked to refrain from strenuous exercise for 24 h prior to HSTs and using a food diary follow a consistent food intake. They were asked to refrain from caffeine and alcohol consumption 12 h before all testing procedures.

As a methodological control participants completed HST2 and HST3 in the same phase of their menstrual cycle (luteal phase), in the active pill portion of the OCP. This was reported by menstrual cycle questionnaire. This detailed the start of the menstrual cycle, premenstrual symptoms and contraceptive medication. This was confirmed by baseline measurement of plasma 17β-estradiol (Table 1).

**TABLE 1:** Mean ± SD plasma 17β-estradiol in HST2 (pre-) and HST3 (post-) STHA in the luteal phases of the menstrual cycle.

Menstrual cycle week (*n* = 8)	3	3
Heat stress test	HST2	HST3
Plasma 17β-estradiol (pg⋅mL^–1^)	29.7 ± 16.4	28.7 ± 8.0

#### Short-Term Heat Acclimation (STHA)

The STHA protocol consisted of five consecutive day’s heat exposure (39.5°C; 60% RH) for 90 min a day, using the controlled hyperthermia technique (Garrett et al., 2009), with permissive dehydration (Garrett et al., 2014). Participants cycled (Monark 824E, Monark Exercise AB, Varberg, Sweden) against a self-selected resistance at 60 rpm attaining a *T*_*re*_ 38.5°C, as quickly as possible and maintained for the 90 min of exposure by regular adjustment of workload. However, an initial workload of 60 watts for the first 5 min duration was the same for all participants at the start of each day’s trial. Elevation of *T*_*re*_ to the same point during heat exposures was to increase workload progressively during the week. The fluid-regulatory hormone aldosterone [aldosterone]_p_, electrolytes (*Na*^+^, *K*^+^, and *Cl*^−^), proteins (total protein [TP]_p_; Albumin [alb]_p_), cortisol [cortisol]_p_ and percentage change in plasma volume (%PV) were measured at rest and end of acclimation bouts on day 1 and day 5. Time (minutes) time to a *T*_*re*_ of 38.5°C and work (J) was recorded on each day of acclimation.

##### Urinary measures

Urine samples were obtained before and after day 1 and day 5 acclimation. Using fresh urine samples, urine specific gravity and urine color were measured using a calibrated refractometer (Uricon-N, Urine specific gravity refractometer, Atago Co., Tokyo, Japan) and urine color chart (Armstrong et al., 1994, 1998), respectively. Urine volume was recorded and urine osmolality was analyzed after the experiment.

##### Blood measures

Plasma for the measurement of the fluid regulatory hormone aldosterone (200 μl) was stored using chilled K-EDTA tubes (1.6 mg⋅ml^–1^). Measurement of aldosterone and cortisol used the Coat-A Count aldosterone procedure. The intra-assay coefficient of variation for aldosterone and cortisol was 8.8 and 12.1%, respectively, for duplicate measures. All samples for a given individual were analyzed within the same assay. Plasma *Na*^+^, *K*^+^, and *Cl*^−^ was analyzed using duplicate colorometric analysis (Cobas Mira Plus, New Jersey, United States).

Changes in the concentration of hemoglobin [Hb] and haematocrit [Hct] were used to determine the relative change in plasma volume described by Dill and Costill (1974). Venous blood samples (5 mL) were taken from an antecubital vein (Vacutainer Precision Glide 21-gauge needle, Becton Dickinson Vacutainer Systems) by phlebotomy without stasis and immediately analyzed – in sexplicate – for [Hb] (Willoughby et al., 2002), (Model OSM3, Radiometer, Copenhagen, Denmark), and [Hct] (using a Hawksley Microhematocrit centrifuge [Sussex, United Kingdom] and a Micro-capillary reader [Damon/IEC Division, Mass, United States]). The percentage change in plasma volume was analyzed from day 1 to 5 of the acclimation regime, during HSTs and calculated using a mathematical equation (Dill and Costill, 1974).

#### Aerobic Fitness Testing and Cardiac Output

Participants performed an incremental ramp exercise test to volitional exhaustion on a treadmill (h/p/Cosmos, Model Pulsar 3p, Traunstein, Germany), for determination of peak oxygen uptake ($\dot{\text{V}}\,{\text{O}}_{2\,\mathrm{peak}}$) and velocities for individualisation of the HSTs. This procedure involved a starting velocity of 5 km/hr with workload increments of 0.1 km/hr every 6 s (1 km/hr/min), until volitional exhaustion. Breath by breath expired air was collected via a metabolic cart system (Cortex Metalyzer 3B, Cortex Biophysic, Leipzig, Germany). Participants RPE (Borg, 1982) and *f*_*c*_ (Polar FS1, Polar Electro, OY, Finland) were recorded every minute. All participants were given verbal encouragement in the latter stages of the incremental test.

Baseline cardiac output was measured using a breath-by-breath cardiac output analyser (Innocor, Innovision, Odense, Denmark). Prior to measurement, calibration of the cardiac output analyser was completed. Each participant had a fresh mouthpiece connected to a bacterial filter (Innovision, Odense, Denmark). A nose clip (Innovision, Odense, Denmark) was then placed over the participant’s nose to prevent any expired air escaping. Participants were instructed to breathe in synchronization (∼5 breaths, ∼15 s) with the on-screen demonstration until measurement was complete.

#### Heat Stress Test (HST)

The HST took place in an environmental chamber (Type SSR 60-20H, Design Environment, Gwent, United Kingdom) set to ambient temperature of 31°C; 50% RH. Pre-exercise urine and blood measure (%PV) were taken prior to entering the chamber. The HST consisted of 9 × 5 min blocks of intermittent exercise on a treadmill (Pulsar 3p, h/p/Cosmos, Traunstein, Germany) and cycle ergometer (Wattbike Ltd., Nottingham, United Kingdom). Each 5-min block consisted of intermittent treadmill running; standing (recovery), walking (50% HRmax), jogging (60% HRmax), low- (70% HRmax), moderate- (85% HRmax) and high-intensity (95% HRmax), ending with a 6-s maximal cycle ergometer sprint. Treadmill velocity changed every 5–11 s and percentage time spent at each velocity was adapted from collegiate level football match play characteristics (Vescovi and Favero, 2014). Sprint characteristics; peak power output (PPO) and mean power output (MPO) were used as performance measures.

Prior to acclimation, as a control trial, two HST’s (HST1 vs. HST2) were performed and separated by 1 week. The post acclimation HST3 was performed within a week (5 ± 2 days) of the final acclimation day to prevent the decay of acclimation (Garrett et al., 2009) and we acknowledge there may be some variability in the magnitude of response (Waldron et al., 2019). To control for menstrual cycle phase the HST2 (Pre-) and HST3 (Post-) STHA trials were performed in week three of the menstrual cycle (luteal phase), in the active pill portion of the OCP for all participants. Baseline measures of plasma 17β-estradiol were taken prior to HST2 and HST3 trials in the luteal phase of the menstrual cycle and there was no difference (*P* = 0.87) observed (Table 1).

##### Body temperature

Core body temperature was measured using a rectal thermistor (U thermistor, Grant Instruments Ltd., Cambridge, United Kingdom) was self-inserted to a depth of 10 cm beyond the anal sphincter. Skin temperature was measured using skin thermistors (Type EUS-U-V5-V2, Grant Instruments Ltd., Cambridge, United Kingdom) placed on four, left sided, sites: chest, bicep, thigh, and calf, secured using micropore tape. Mean skin temperature (${\bar{T}}_{s\,k}$) (Ramanathan, 1964) and mean body temperature (${\bar{T}}_{b}$) (Sawka et al., 1996) were measured. Temperature data was recorded at 1-min intervals on a portable data logger (2020 series data logger, Grant Instruments Ltd., Cambridge, United Kingdom).

##### Data analysis

Sample size was based upon results from the previous limited research on females (Mee et al., 2015; Kirby et al., 2019), using our permissive dehydration protocol during STHA (Garrett et al., 2014). Where a statistical difference was observed in primary outcomes. The stress response of dependent measures in STHA and HSTs were analyzed for normal distribution by using the Shapiro–Wilk and the Brown–Forsythe test determined equal variance. All data were normally distributed and two-way repeated measures ANOVA was used to determine main effects between day 1 and 5 acclimation, pre vs post HSTs and interaction over time for all dependent measures. Pairwise multiple comparison procedures were analyzed using post-hoc Bonferroni correction *t*-tests when appropriate. The change in thermal markers on day 1 and day 5 of acclimation were analyzed using paired *t*-test analysis. Work output from day 1 to day 5 of acclimation was analyzed using one-way analysis of variance ANOVA, with repeated measures and Bonferroni correction *t*-tests to isolate differences between days. Data is reported for ten moderately trained females unless otherwise stated. Where appropriate data is reported as mean differences ± SD with 95% confidence intervals (95% CI) and the magnitude of effect using Cohen’s *d* effect sizes (where 0.2–0.59 small; 0.6–1.19 moderate; 1.2–1.99 large; 2.0–4.0 very large).

## Results

All ten participants completed the 5-day STHA protocol and three HSTs (HST1; HST2; HST3). The HST1 versus HST2 was a control trial taken 1 week apart with no intervention. The HST2 versus HST3 with the STHA intervention took place in week 3 of the menstrual cycle (luteal phase) for all ten participants. Due to issues with venepuncture measures blood parameters were analyzed for eight participants only. Similarly, eight participants had baseline plasma 17β-estradiol measured before HST2 and HST3 trials in the luteal phase.

### Acclimation

#### Thermal Stress and Strain

**FIGURE 2:**
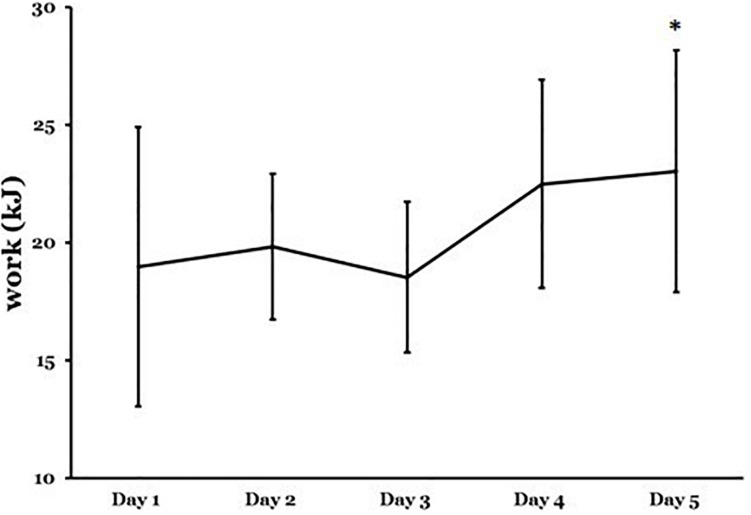
Work output on the first day (Day 1) to the last day (Day 5) of acclimation after 90-min heat exposure. Data are mean ±SD are for ten moderately trained females. Significant difference ^∗^*p* < 0.05; Day 1 to the last day of acclimation analyzed using one-way analysis of variance (ANOVA) with repeated measures and Bonferroni correction *t*-tests to isolate differences between days.

Thermal stress and strain from days 1 to 5 of heat acclimation are presented in Table 2 and work completed in Figure 2.

**TABLE 2:** Thermal stress and strain on the first (Day 1) and last day (Day 5) of short-term heat acclimation (STHA) for ten moderately trained females.

	**Day 1**	**Day 5**	***P*-value**
*T*_*a*_ (°C)	39.6 ± 0.1	39.7 ± 0.2	0.14
RH (%)	60.0 ± 0.2	60.1 ± 0.1	0.72
Mean *f*_*c*_ (b⋅min^–1^)	144 ± 22	141 ± 19	0.11
Mean *T*_*re*_ (°C)	38.29 ± 0.46	38.24 ± 0.47	0.24
Time to *T*_*re*_ 38.5°C (min)	36.70 ± 6.36	44.62 ± 11.04	**0.04**
Work (KJ)	18.98 ± 5.94	23.03 ± 5.14	**0.02**
Body mass change (%)	−1.7 ± 0.6	−1.8 ± 0.7	0.80
%PV change	0.9 ± 13.1	0.7 ± 12.0	0.98

*Ambient temperature (*T*_*a*_), RH cardiac frequency (*f*_*c*_), rectal temperature (*T*_*re*_), time to *T*_*re*_ 38.5°C and work on day 1 and 5 of acclimation undertaken with no fluid intake. Data presented as mean ±SD for ten female participants. Significant differences by paired *t*-test are shown in bold.*

Measures of ambient temperature (*T*_*a*_) and RH indicated that the thermal stress was similar on day 1 and 5 of acclimation. Similarly, the thermal strain was consistent between days illustrated by mean cardiac frequency (*f*_*c*_) and rectal temperature (*T*_*re*_) responses. Time to 38.5°C was longer on day 5 cf. day 1 (Table 2; *P* = 0.04). Therefore, less work was performed on day 1 cf. day 5 (Table 2 and Figure 2; *P* = 0.02).

#### Urinary Measures

To determine hydration status urine color (color_u_), urine osmolality (osm_u_), urine specific gravity (SG_u_) and body mass were measured at rest and 90 min, on day 1 and 5 of acclimation (Table 3). There was no main effect (*P* > 0.05) and interaction across time (*P* > 0.05) for color_u_, osm_u_, SG_u_ and body mass on day 1 and 5 of STHA.

**TABLE 3:** Urinary measures of hydration (color_u_, osm_u_, SG_u_) and nude body mass, at rest and end-exercise, on day 1 and 5 of short-term heat acclimation.

	**Day 1:rest**	**Day 1:end**	**Day 5:rest**	**Day 5:end**
Color_u_ (units)	2 ± 1	4 ± 2	3 ± 1	4 ± 2
osm_u_ (mOsm/kg)	379 ± 292	447 ± 181	379 ± 267	396 ± 271
SG_u_ (units)	1.008 ± 0.007	1.012 ± 0.007	1.008 ± 0.006	1.010 ± 0.008
Body mass (kg)	62.3 ± 9.9	61.2 ± 9.8	62.5 ± 9.8	61.4 ± 9.7

*Data presented as mean ±SD for ten female participants. A two-way repeated measures ANOVA and post-hoc Bonferroni correction *t*-tests when appropriate was used to determine the differences from rest to end-heat exposure, on day 1 and 5 of short-term heat acclimation.*

#### Blood Measurements

Blood measures and percentage change on the first day (Day 1) to the last day (Day 5) of acclimation after 90-min heat exposure are presented in Table 4.

**TABLE 4:** Blood measures and percentage change from rest to end-exposure on the first day (Day 1) versus the last day (Day 5) of acclimation after 90-min heat exposure.

	**[aldo]_p_**	**[Na^+^]_p_**	**[TP]_p_**	**[alb]_p_**	**[cortisol]_p_**
	**(pg⋅mL^–1^)**	**(mmol⋅L^–1^)**	**(mg⋅mL^–1^)**	**(mg⋅mL^–1^)**	**(ug⋅dl^–1^)**
**Day 1**					
**Acclimation**					
Rest	216 ± 131	140 ± 2	72.8 ± 3.2	670 ± 36	172 ± 63
End	417 ± 99	141 ± 1	78.3 ± 3.0	716 ± 33	307 ± 47^∗^
%Change	48%	1%	7%	6%	44%
**Day 5**					
**Acclimation**					
Rest	187 ± 64	139 ± 1	71.6 ± 4.8	666 ± 41	190 ± 47
End	332 ± 143	142 ± 2	77.6 ± 5.7	717 ± 52	200 ± 67
%Change	44%	2%	8%	7%	5%

*Data are mean ±SD for eight moderately trained females. A two-way repeated measures ANOVA and post-hoc Bonferroni correction *t*-tests when appropriate was used to determine the differences from rest to end-heat exposure, on day 1 and 5 of short-term heat acclimation. ^∗^*P* < 0.05 at rest versus end-heat exposure (90 min) on day 1 and 5.*

There was no main effect for [aldo]_p_ between day 1 and 5 (*F* = 1.583; *P* = 0.25) or interaction across time (*F* = 0.755; *P* = 0.41). Similarly, there was no main effect between day 1 and 5 for [Na^+^]_p_ (*F* = 1.106; *P* = 0.32) or interaction across time (*F* = 0.983; *P* = 0.36). The measure [TP]_p_ demonstrated no main effect between day 1 and 5 (*F* = 0.93; *P* = 0.37) or interaction across time (*F* = 0.198; *P* = 0.67). There was no main effect between day 1 and 5 (*F* = 0.0618; *P* = 0.81) for [alb]_p_ or interaction across time (*F* = 0.50; *P* = 0.50). In contrast, there was a significant main effect between day 1 and 5 for [cortisol]_p_ (*F* = 15.303; *P* = 0.01), and interaction across time (*F* = 10.775; *P* = 0.02). Bonferroni-corrected post-hoc comparisons showed a significant difference between pre and post measures within day 1 (*P* = 0.002) but not on day 5 (*P* = 0.57).

#### Heat Stress Test

Measurements were taken at rest and across the 45 min in HSTs. Data is presented for ten female participants unless otherwise stated.

#### Control Study

The HST1 versus HST2 was a control trial taken 1 week apart with no intervention. There was a limited change for *T*_*re*_, ${\bar{T}}_{s\,k}$, ${\bar{T}}_{b}$, *f*_*c*_, and %PV (*P* > 0.05). Similarly, in the sprint performance test the PPO and MPO demonstrated limited change (*P* > 0.05).

#### Intervention Study

The HST2 trial took place 1 week before the STHA (5-days), with no fluid intake intervention. The post HST3 occurred within 7-days of the last acclimation.

##### Body temperatures

**FIGURE 3:**
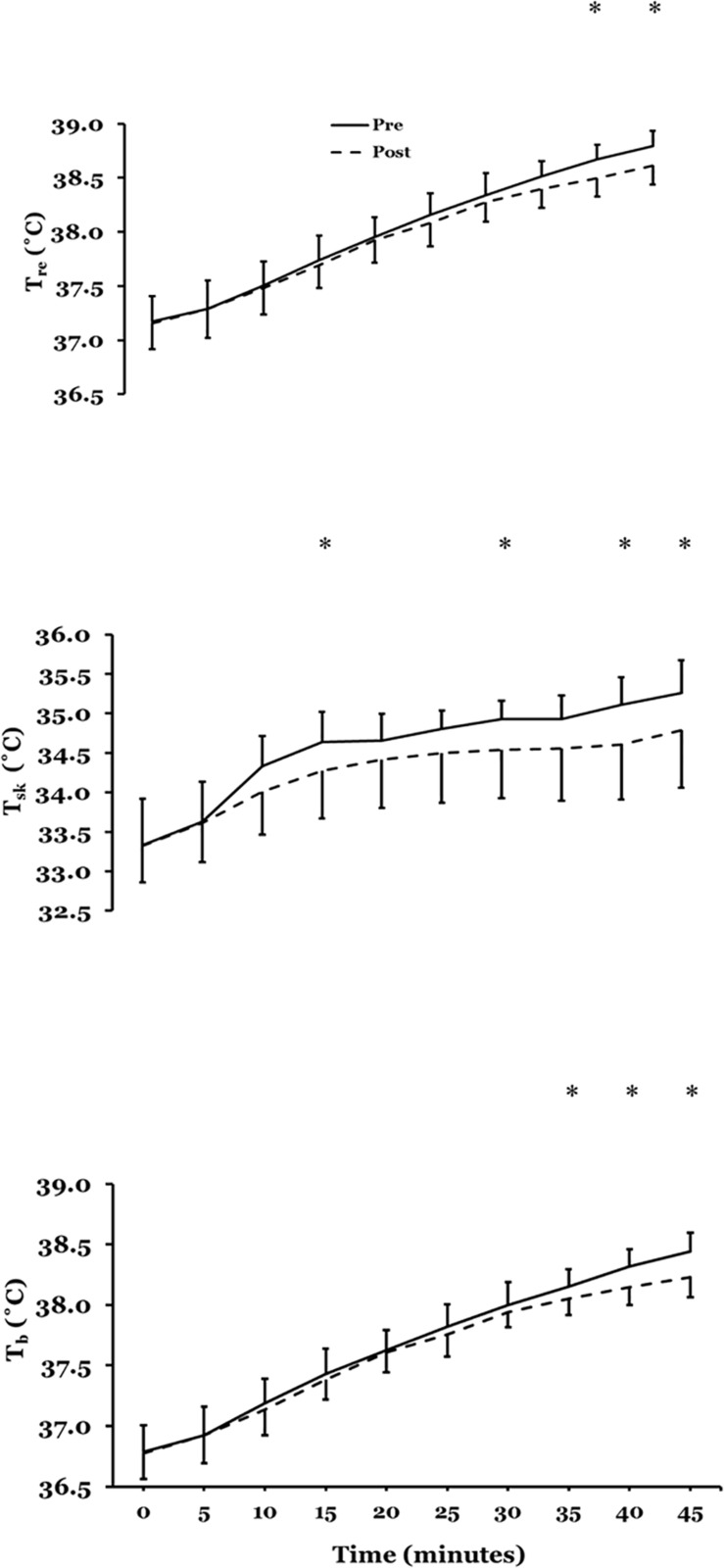
Mean ± SD rectal temperature (*T*_*re*_) (upper plate), mean skin temperature (${\bar{T}}_{s\,k}$) (mid plate) and mean body temperature (${\bar{T}}_{b}$) (lower plate), pre- to post acclimation in hot conditions (31°C; 50% RH; *n* = 10). ^∗^*P* < 0.05 post-hoc Bonferroni correction *t*-tests.

Figure 3 presents mean ± SD rectal temperature (*T*_*re*_), mean skin temperature (${\bar{T}}_{s\,k}$) and mean body temperature (${\bar{T}}_{b}$), pre- to post acclimation in hot conditions (31°C; 50% RH; *n* = 10).

There was no main effect for *T*_*re*_(*F* = 1.411; *P* = 0.27) after STHA but there was a significant interaction across time (*F* = 2.991; *P* = 0.004). Bonferroni-corrected post-hoc comparisons showed a significant mean difference at 40 (*P* = 0.01) and 45 min (*P* = 0.007). At 45 min *T*_*re*_ reduced by 0.20°C (95%CI −0.30 to −0.10°C; *d* = 0.77: Moderate). There was a significant main effect for ${\bar{T}}_{s\,k}$ (*F* = 5.252; *P* = 0.05) after STHA and interaction across time (*F* = 4.689; *P* = 0.001). Bonferroni-corrected post-hoc comparisons showed a significant mean difference at 15 (*P* = 0.043), 30 (*P* = 0.03); 35 (*P* = 0.004); 40 (*P* = 0.009) and 45 min (*P* = 0.009). The reduction in ${\bar{T}}_{s\,k}$ at 45 min was −0.50 (−0.90 to −0.10°C; *d* = 0.80: Moderate). There was no main effect for ${\bar{T}}_{b}$ (*F* = 4.419; *P* = 0.07) after STHA but there was a significant interaction across time (*F* = 3.942; *P* = 0.001). Bonferroni-corrected post-hoc comparisons showed a significant mean difference at 35 (*P* = 0.01), 40 (*P* = 0.002) and 45 min (*P* = 0.001). At 45 min ${\bar{T}}_{b}$ reduced by −0.25 (−0.35 to −0.15°C; *d* = 0.92: Moderate).

##### Cardiac frequency and percentage change in plasma volume (%PV)

There was a significant main effect for cardiac frequency (*F* = 7.702; *P* = 0.02) after STHA and interaction across time (*F* = 2.485; *P* = 0.02). Bonferroni-corrected post-hoc comparisons showed a significant mean difference at rest (*P* = 0.001), 5 (*P* = 0.04) and 45 min (*P* = 0.003). Cardiac frequency reduced at rest (−13; −18 to −7 b⋅min^–1^; *d* = 1.04: Moderate) and at 45-min (−8; −16 to −1 b⋅min^–1^; *d* = 1.11: Moderate). There was an increase in %PV from baseline post STHA by 7.0% (−0.4 to 14.5%, *d* = 1.27: Large).

##### Psychophysiological

There was a significant main effect for thermal comfort (*F* = 27.156; *P* = 0.001) after STHA and interaction across time (*F* = 3.378; *P* = 0.001). Bonferroni-corrected post-hoc comparisons showed a significant mean difference from 10 to 45 min (*P* < 0.05). Thermal comfort reduced at 45 min by −1 (−1.5 to −0.5 units; *d* = 0.89: Moderate). There was a significant main effect for thermal sensation (*F* = 19.462; *P* = 0.002) after STHA and interaction across time (*F* = 4.533; *P* = 0.001). Bonferroni-corrected post-hoc comparisons showed a significant mean difference at 0 to 10 and 20 to 45 min (*P* < 0.05). Thermal sensation reduced at 45 min by −1 (−1.5 to −0.0 units; *d* = 0.67: Moderate). There was a significant main effect for RPE (*F* = 5.831; *P* = 0.04) after STHA and interaction effect across time (*F* = 2.853; *P* = 0.006). Bonferroni-corrected post-hoc comparisons showed a significant mean difference 20 (*P* = 0.04), 25 (*P* = 0.04) 30 (*P* = 0.02), 40 (*P* = 0.01) and 45 min (*P* = 0.01). RPE decreased at 45 min by −2 (−4 to 0 units; *d* = 0.70: Moderate).

##### Repeated sprint performance

**FIGURE 4:**
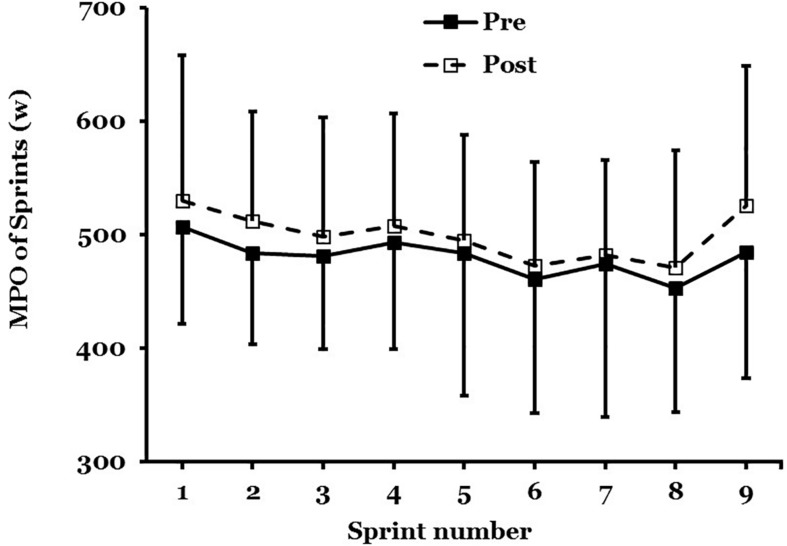
Mean ± SD of mean power output (MPO) in maximal sprint performance, pre- to post acclimation in hot conditions (31°C; 50% RH; *n* = 9).

The PPO and MPO were measured across all nine, 6-s maximal sprints in the 45-min protocol (Figure 4).

There was no main effect for PPO (*F* = 1.458; *P* = 0.26) after STHA or significant interaction effect across all nine sprints (*F* = 0.397; *P* = 0.91). There was not a main effect for MPO (*F* = 3.064; *P* = 0.12) after STHA but the interaction across all nine sprints was close to significance (*F* = 0.296; *P* = 0.06). The MPO increased across all nine maximal sprints by 56W (−26 to 139W; *d* = 0.69: Moderate; *n* = 9).

## Discussion

### Effectiveness of Short-Term Heat Acclimation

The adaptations from short-term (5-d) heat acclimation with no fluid intake during acclimation, using the controlled hyperthermia technique, reduced exercising cardiovascular strain in females controlling for menstrual cycle phase. The cardiovascular stability was due to increased heat loss rather than lower heat content (∼resting core temperature), at the time of day of testing HSTs. This concurs with our previous work that has used no fluid intake during acclimation but with moderately (Garrett et al., 2009) and highly trained males (Garrett et al., 2012). However, it is in contrast with the limited work on STHA with females, using controlled-hyperthermia with permissive dehydration (Mee et al., 2015; Kirby et al., 2019).

### Adaptation to Exercise in the Heat and Menstrual Cycle Phase

The post acclimation HST3 was performed within a week of the final acclimation day to prevent the decay of acclimation (Garrett et al., 2009). It has long been recognized that the menstrual cycle plays a significant role in athletic performance (Avellini et al., 1979; Tenaglia et al., 1999; Janse de Jonge et al., 2012). Therefore, to control for menstrual cycle phase the HST2 (Pre-) and HST3 (Post-) STHA trials were performed in week 3 of menstrual cycle (luteal phase), with all participants using oral contraceptive pills (combined). This was determined by menstrual cycle questionnaire and baseline measures of plasma 17β-estradiol. This was measured prior to HST2 and HST3 intervention trials and there was no statistical difference observed (Table 1). It has previously been reported that heat adaptation in females is not affected by menstrual cycle phase (Lei et al., 2017; Lei and Mundel, 2018) or the use of oral contraceptive pill (Armstrong et al., 2005). However, menstrual cycle phase and the associated changes in female sex hormones can influence core temperature (Inoue et al., 2005), the overall thermoregulatory set point range (Charkoudian and Stachenfeld, 2016) but a limited effect on whole body heat loss has been reported (Notley et al., 2018).

The present results using a female cohort and controlling for menstrual cycle phase, undergoing STHA of daily controlled hyperthermia with no fluid intake, demonstrated that the participants experienced adaptation to the heat. This was indicated by the characteristic features of acclimation. A decrease in *T*_*re*_ by −0.2°C (Figure 3; top panel), ${\bar{T}}_{s\,k}$ by −0.5°C (Figure 3; mid panel) and ${\bar{T}}_{b}$ by −0.25 (Figure 3; lower panel) was observed. Similar body temperature measures have previously been reported by the author, using the hyperthermia-control technique but with male participants (Garrett et al., 2009, 2012, 2014). In contrast, the research group of Mee et al. (2015) reported that the employment of the controlled hyperthermia model with permissive dehydration successfully attenuated *T*_*re*_ during a 30-min run in the heat after 5 days in males (−0.39 ± 0.36°C) but not in females (−0.07 ± 0.18°C). Yet after a further 5 days of acclimation the females *T*_*re*_ response was similar by 0.48 ± 0.27°C (Mee et al., 2015). This indicates that a female population requires a longer-term intervention than the 5-days STHA we employed (Mee et al., 2015). Similarly, Kirby et al. (2019) determined nine-, but not 4-days heat acclimation improves self-paced endurance performance in females, using hyperthermia control with permissive dehydration (Kirby et al., 2019). However, this was over a shorter 4-day STHA. Furthermore, in these two studies there were method differences with the present work. The exercise protocols were short in duration (∼30 min), self-paced/fixed speed trials and importantly menstrual OCP phase was not controlled.

End-exercise  *f*_*c*_ decreased by 8 b⋅min^–1^. Increased cardiovascular stability is recognized as one of the most rapidly occurring adaptations to the heat (Garrett et al., 2009, 2011). Furthermore, a 7.0% PV expansion from baseline was observed in this study. Previous research suggests intravascular fluid expansion resulting from such increases in colloid-osmotic pressure (Senay et al., 1976; Senay, 1979) explains a majority of PV expansion (Goto et al., 2010). Therefore, this suggests that the PV expansion may have contributed to the greater cardiovascular stability that was observed. This is similar to the findings of Pethick et al. (2018) who demonstrated induced plasma volume expansion in a female cohort following 5-days high-intensity heat acclimation (Pethick et al., 2018).

### Repeated Sprint Performance

This study demonstrated that an increase in MPO was close to significance across all nine maximal sprints after STHA of 5-days (Figure 4). This improvement in intermittent performance is supported by Sunderland et al. (2008) who developed a heat acclimation protocol (4-days) for female team sports (Sunderland et al., 2008). They reported a reduced rate of rise in rectal temperature and a 33% improvement in distance run during a repeated shuttle run performance test after STHA in a female cohort. From a practical perspective, an improvement in sprint performance is a valuable asset in team sport situations. Work-rate during team sports matches are largely determined by the oppositions playing style of the opposing team and individuals (Ozgunen et al., 2010), the ability to maintain repeated sprint performance can determine when a games player gets to the ball first and outrun the opposition.

### Fluid Regulation Response to Repeated Heat Stress

In the present study, participants experienced the same thermal load and this is the basis of using the controlled hyperthermia technique for heat acclimation. Individual’s experienced a mild hypohydration of ∼2% body mass (Table 2). This is similar to the imposed hypohydration administered by Judelson and colleagues, who reported a modification in the hormonal and metabolic response to resistance exercise, influencing the post-exercise circulatory milieu (Judelson et al., 2008). The research design of this study is supported by recommendations from earlier work with females on sex hormones and fluid regulation by the Stachenfeld research group (Stachenfeld et al., 1999; Stachenfeld, 2008).

### Fluid Regulatory Hormones, Electrolytes and Plasma Volume Expansion

In the present study, after 90-min exercise [aldo]_p_ did not significantly increase across acclimation bouts (Table 4) and this is in contrast to what has previously been reported (Judelson et al., 2008). The principal effects of aldosterone are the retention of *Na*^+^ and therefore water from the urine output to maintain extracellular fluid volume and thus blood volume. However, in the present study, an exercise-induced response of increased [*Na*^+^]_p_ was not clearly evident after the no fluid intake acclimation regime (Table 4). Therefore, this is in contrast with previous findings (Brandenberger et al., 1989; Francesconi et al., 1993; Allsopp et al., 1998) who reported a strong relationship between increased *Na*^+^ with [aldo]_p_ response. In the present study, using the ΔPV (Dill and Costill, 1974) technique, there was an acclimation induced increase in resting %PV across HSTs by 7.0 ± 6% in the present study. This is similar to Pethick et al. (2018) who successfully induced plasma volume expansion in a female cohort, following 5-days high-intensity heat acclimation (Pethick et al., 2018).

### Stress Hormone Response

In the present study the time to reach 38.5°C significantly increased (21.6%) from day 1 to 5 resulting in an associated increase in work (21.3%) (Table 2). Mean time to reach 38.5°C has been shown to be longer during STHA, using the controlled hyperthermia technique for females (51 ± 7 min), in comparison with males (48 ± 9 min) (Mee et al., 2015). Similarly, in the present study, a much larger % difference in time to 38.5°C was observed in comparison with previous studies using male cohorts with the same protocol (Garrett et al., 2009, 2012, 2014; Neal et al., 2015). The stress hormone, cortisol, significantly increased during acclimation on day 1 but this response was not observed on day 5. Despite a greater time to 38.5°C and more work being completed, hence, indicating a heat adaptive response (Table 4). This agrees with previous observations on male cohorts suggesting heat acclimation reduces cortisol levels during exercise in the heat (Francesconi et al., 1983; Armstrong et al., 1989) but such findings are not universal (Finberg and Berlyne, 1977; Sunderland et al., 2008).

## Limitations and Future Directions

In order to standardize menstrual cycle phase each female participant used a monophasic, oral contraceptive pill (OCP) but a potential limitation was that the dose of hormone concentration differed between individuals depending on their specific medication.

For future directions, information is limited on the physiological mechanisms of fluid regulation in females, following STHA. Therefore, a comparison of euhydration versus dehydration STHA, may provide a greater understanding of this area. To the authors knowledge, our earlier work (Garrett et al., 2014) is the only study to have done this but with male participants.

## Conclusion

In summary, this work has established the effectiveness of STHA for 5 days, using the controlled-hyperthermia technique with no fluid intake (Garrett et al., 2009, 2012, 2014), on intermittent activity in hot environments with a female cohort, controlling for menstrual cycle phase. The current research suggests these methods of heat acclimation in a female cohort enhances thermoregulation and cardiovascular stability during intermittent exercise in the heat. These improvements may provide protection from exertional heat related illnesses associated with exercise performance. This work adds to the limited body of literature available and this is particularly important given the 2020 Olympics will be held in the hot and humid conditions of Tokyo in Japan.

## Data Availability Statement

The datasets generated for this study are available on request to the corresponding author.

## Ethics Statement

The studies involving human participants were reviewed and approved by the University of Hull Ethics Committee. The patients/participants provided their written informed consent to participate in this study.

## Author Contributions

AG conceived and designed the research. JG, ED, VB, and JS conducted the experiments. JB, HJ, DG-S, and RB contributed to the blood handling and analysis. AG and GA analyzed the data. AG and JG wrote the manuscript. All authors read and approved the manuscript.

## Conflict of Interest

The authors declare that the research was conducted in the absence of any commercial or financial relationships that could be construed as a potential conflict of interest.

## Abbreviations

*f*_*c*_: cardiac frequency (b ⋅ min^–1^)
$\dot{Q}$: cardiac output (L ⋅ min^–1^)
HST: heat stress test
$\dot{\text{V}}\,{\text{O}}_{2\,\max}$: maximum oxygen uptake (L ⋅ min^–1^or mL ⋅ kg^–1^ ⋅ min^–1^)
MPO: mean power output (W)
${\bar{T}}_{b}$: mean body temperature (°C)
${\bar{T}}_{s\,k}$: mean skin temperature (°C)
$\dot{\text{V}}\,{\text{O}}_{2}$: oxygen consumption (L ⋅ min^–1^ or mL ⋅ kg^–1^ ⋅ min^–1^)
$\dot{\text{V}}\,{\text{O}}_{2}\,\mathrm{peak}$: peak oxygen uptake (L ⋅ min^–1^or mL ⋅ kg^–1^ ⋅ min^–1^)
PPO: peak power output (W)
%PV: percentage plasma volume (%)
[alb]_p_: plasma albumin (mg ⋅ mL^–1^)
[aldo]_p_: plasma aldosterone (pg ⋅ mL^–1^)
[cortisol]_p_: plasma cortisol (ug ⋅ dl^–1^)
[Na^+^]_p_: plasma sodium (mmol ⋅ L^–1^)
TP_p_: plasma total protein (mg ⋅ mL^–1^)
*T*_*re*_: rectal temperature (°C)
RH: relative humidity (%)
STHA: short-term heat acclimation
color_u_: urine color (units)
osm_u_: urine osmolality (mOsm/kg)
SG_u_: urine specific gravity (units).

## References

[B1] Akerman A. P. Tipton M. Minson C. T. Cotter J. D. (2016). Heat stress and dehydration in adapting for performance: good, bad, both, or neither? *Temperature* 3 412–436. 10.1080/23328940.2016.1216255 28349082 PMC5356617

[B2] Allsopp A. J. Sutherland R. Wood P. Wooton S. A. (1998). The effect of sodium balance on sweat sodium secretion and plasma aldosterone concentration. *Eur. J. Appl. Physiol.* 78 516–521. 10.1007/s004210050454 9840406

[B3] Armstrong L. E. Francesconi R. P. Kraemer W. J. Leva N. De Luca J. P. Hubbard R. W. (1989). Plasma cortisol, renin, and aldosterone during an intense heat acclimation program. *Int. J. Sports Med.* 10 38–42. 10.1055/s-2007-1024871 2649446

[B4] Armstrong L. E. Herrera Soto J. A. Hacker F. T. Casa D. J. Kavouras S. A. Maresh C. M. (1998). Urinary indices during dehydration, exercise, and rehydration. *Int. J. Sport Nutr.* 8 345–355. 10.1123/ijsn.8.4.345 9841955

[B5] Armstrong L. E. Maresh C. M. Castellani J. W. Bergeron M. F. Kenefick R. W. LaGasse K. E. (1994). Urinary indices of hydration status. *Int. J. Sport Nutr.* 4 265–279. 10.1123/ijsn.4.3.265 7987361

[B6] Armstrong L. E. Maresh C. M. Keith N. R. Elliott T. A. Van Heest J. L. Scheet T. P. (2005). Heat acclimation and physical training adaptations of young women using different contraceptive pills. *Am. J. Physiol. Endocrinol. Metab.* 288 E868–E875. 15598669 10.1152/ajpendo.00434.2004

[B7] Avellini B. A. Kamon E. Krajewski J. T. (1979). Physiological responses of physically fit men and women to acclimation to humid heat. *DTIC Doc.* 49 254–261. 10.1152/jappl.1980.49.2.254 7400008

[B8] Borg G. A. (1982). Psychophysical bases of perceived exertion. *Med. Sci. Sports Exerc.* 14 377–381. 7154893

[B9] Brandenberger G. Candas V. Follenius M. Kahn J. M. (1989). The influence of initial state of hydration on endocrine responses to exercise in the heat. *Eur. J. Appl. Physiol.* 58 674–679. 10.1007/bf00418516 2543562

[B10] Bruinvels G. Burden R. Brown N. Richards T. Pedlar C. (2016). The Prevalence and Impact of Heavy Menstrual Bleeding (Menorrhagia) in Elite and Non-Elite Athletes. *PLoS One* 11:e0149881. 10.1371/journal.pone.0149881 26901873 PMC4763330

[B11] Charkoudian N. Johnson J. M. (1997). Modification of active cutaneous vasodilation by oral contraceptive hormones. *J. Appl. Physiol.* 83 2012–2018. 10.1152/jappl.1997.83.6.2012 9390975

[B12] Charkoudian N. Stachenfeld N. (2016). Sex hormone effects on autonomic mechanisms of thermoregulation in humans. *Auton. Neurosci.* 196 75–80. 10.1016/j.autneu.2015.11.004 26674572

[B13] Constantini N. W. Dubnov G. Lebrun C. M. (2005). The menstrual cycle and sport performance. *Clin. Sports Med.* 24:e51-82. 15892917 10.1016/j.csm.2005.01.003

[B14] Daanen H. A. Herweijer J. A. (2015). effectiveness of an indoor preparation program to increase thermal resilience in elderly for heat waves. *Build. Environ.* 83 115–119. 10.1016/j.buildenv.2014.04.010

[B15] Dill D. B. Costill D. L. (1974). Calculation of percentage changes in volumes of blood, plasma, and red cells in dehydration. *J. Appl. Physiol.* 37 247–248. 10.1152/jappl.1974.37.2.247 4850854

[B16] Finberg J. P. Berlyne G. M. (1977). Modification of renin and aldosterone response to heat by acclimatization in man. *J. Appl. Physiol. Respir. Environ. Exerc. Physiol.* 42 554–558. 10.1152/jappl.1977.42.4.554 863817

[B17] Francesconi R. N. Amstrong L. E. Leva N. M. Moore R. J. Szlyk P. C. Matthew W. T. (1993). *Endocrinological Responses to Dietary Salt Restriction During Heat Acclimation. Nutritional Needs in Hot Environments.* Washington, D.C: National Academy Press, 259–275.

[B18] Francesconi R. P. Sawka M. N. Pandolf K. B. (1983). Hypohydration and heat acclimation: plasma renin and aldosterone during exercise. *J. Appl. Physiol. Respir. Environ. Exerc. Physiol.* 55 1790–1794. 10.1152/jappl.1983.55.6.1790 6363364

[B19] Garrett A. T. Creasy R. Rehrer N. J. Patterson M. J. Cotter J. D. (2012). Effectiveness of short-term heat acclimation for highly trained athletes. *Eur. J. Appl. Physiol.* 112 1827–1837. 10.1007/s00421-011-2153-3 21915701

[B20] Garrett A. T. Goosens N. G. Rehrer N. J. Patterson M. J. Cotter J. D. (2009). Induction and decay of short-term heat acclimation. *Eur. J. Appl. Physiol.* 107 659–670. 10.1007/s00421-009-1182-7 19727796

[B21] Garrett A. T. Goosens N. G. Rehrer N. J. Patterson M. J. Harrison J. Sammut I. (2014). Short-term heat acclimation is effective and may be enhanced rather than impaired by dehydration. *Am. J. Hum. Biol.* 26 311–320. 10.1002/ajhb.22509 24469986

[B22] Garrett A. T. Rehrer N. J. Patterson M. J. (2011). Induction and decay of short-term heat acclimation in moderately and highly trained athletes. *Sports Med.* 41 757–771. 10.2165/11587320-000000000-00000 21846164

[B23] Gerrett N. Kingma B. R. M. Sluijter R. Daanen H. A. M. (2019). Ambient conditions prior to Tokyo 2020 olympic and paralympic games: considerations for acclimation or acclimatization strategies. *Front. Physiol.* 10:414. 10.3389/fphys.2019.00414 31068829 PMC6491848

[B24] Gibson O. R. Mee J. A. Tuttle J. A. Taylor L. Watt P. W. Maxwell N. S. (2015). Isothermic and fixed intensity heat acclimation methods induce similar heat adaptation following short and long-term timescales. *J. Therm. Biol.* 4 55–65. 10.1016/j.jtherbio.2015.02.005 25774027

[B25] Goto M. Okazaki K. Kamijo Y. Ikegawa S. Masuki S. Miyagawa K. (2010). Protein and carbohydrate supplementation during 5-day aerobic training enhanced plasma volume expansion and thermoregulatory adaptation in young men. *J. Appl. Physiol*. 109 1247–1255. 10.1152/japplphysiol.00577.2010 20689095

[B26] Grucza R. Pekkarinen H. Titov E. K. Kononoff A. Hänninen O. (1993). Influence of the menstrual cycle and oral contraceptives on thermoregulatory responses to exercise in young women. *Eur. J. Appl. Physiol. Occup. Physiol.* 67 279–285. 10.1007/bf00864229 8223544

[B27] Hessemer V. Zeh A. Brück K. (1986). Effects of passive heat adaptation and moderate sweatless conditioning on responses to cold and heat. *Eur. J. Appl. Physiol. Occup. Physiol.* 55 281–289. 10.1007/bf02343800 3732255

[B28] Inoue Y. Tanaka Y. Omori K. Kuwahara T. Ogura Y. Ueda H. (2005). Sex-and menstrual cycle-related differences in sweating and cutaneous blood flow in response to passive heat exposure. *Eur. J. Appl. Physiol.* 94 232–332. 15729550 10.1007/s00421-004-1303-2

[B29] Janse de Jonge X. A. Thompson M. W. Chuter V. H. Silk L. N. Thom J. M. (2012). Exercise performance over the menstrual cycle in temperate and hot, humid conditions. *Med. Sci. Sports Exerc.* 44 2190–2198. 10.1249/MSS.0b013e3182656f13 22776870

[B30] Judelson D. A. Maresh C. M. Yamamoto L. M. Farrell M. J. Armstrong L. E. Kraemer W. J. (2008). Effect of hydration state on resistance exercise-induced endocrine markers of anabolism, catabolism, and metabolism. *J. Appl. Physiol.* 105 816–824. 10.1152/japplphysiol.01010.2007 18617629

[B31] Kampmann B. Brode P. Schutte M. Griefahn B. (2008). Lowering of resting core temperature during acclimation is influenced by exercise stimulus. *Eur. J. Appl. Physiol.* 104 321–327. 10.1007/s00421-007-0658-6 18193268

[B32] Kirby N. V. Lucas S. J. E. Lucas R. A. I. (2019). Nine-, but not four-days heat acclimation improves self-paced endurance performance in females. *Front. Physiol.* 10:539 10.3389/fphys.2019.00539 PMC6532023 31156449

[B33] Kolka M. A. Stephenson L. A. (1989). Control of sweating during the human menstrual cycle. *Eur. J. Appl. Physiol. Occup. Physiol.* 58 890–895. 10.1007/bf02332224 2767071

[B34] Kolka M. A. Stephenson L. A. (1997). Interaction of menstrual cycle phase, clothing resistance and exercise on thermoregulation in women. *J. Therm. Biol.* 22 137–141. 10.1016/s0306-4565(97)00003-x

[B35] Lebrun C. M. McKenzie D. C. Prior J. C. Taunton J. E. (1995). Effects of menstrual cycle phase on athletic performance. *Med. Sci. Sports Exerc.* 27 437–444. 7752873

[B36] Lei T. H. Mundel T. (2018). Humid heat stress affects trained female athletes more than does their menstrual phase. *Temp. Vol.* 5 202–204. 10.1080/23328940.2018.1436394 30377639 PMC6205064

[B37] Lei T.-H. Stannard S. R. Perry B. G. Schlader Z. J. Cotter J. D. Mu T. (2017). Influence of menstrual phase and arid vs. humid heat stress on autonomic and behavioural thermoregulation during exercise in trained but unacclimated women. *J. Physiol.* 595 2823–2837. 10.1113/JP273176 27900769 PMC5407968

[B38] Mee J. A. Gibson O. R. Doust J. Maxwell N. S. (2015). A comparison of males and females’ temporal patterning to short- and long-term heat acclimation. *Scand. J. Med. Sci. Sports* 25(Suppl. 1), 250–258. 10.1111/sms.12417 25943676

[B39] Neal R. A. Corbett J. Massey H. C. Tipton M. J. (2015). Effect of short-term heat acclimation with permissive dehydration on thermoregulation and temperate exercise performance. *Scand. J. Med. Sci. Sports* 26 875–884. 10.1111/sms.12526 26220213

[B40] Neal R. A. Massey H. C. Tipton M. J. Young J. S. Corbett J. (2016). Effect of permissive dehydration on induction and decay of heat acclimation, and temperate exercise performance. *Front. Physiol.* 7:564. 10.3389/fphys.2016.00564 27932993 PMC5120118

[B41] Notley S. R. Dervis S. M. Poirier M. P. Kenny G. P. (2018). Menstrual cycle phase does not modulate whole-body heat loss during exercise in hot dry conditions. *J. Appl. Physiol.* 126 286–293. 10.1152/japplphysiol.00735.2018 30496713 PMC6397413

[B42] Ozgunen K. T. Kurdak S. S. Maughan R. J. Zeren C. Korkmaz S. Yazici Z. (2010). Effect of hot environmental conditions on physical activity patterns and temperature response of football players. *Scand. J. Med. Sci. Sports* 20(Suppl. 3), 140–147. 10.1111/j.1600-0838.2010.01219.x 21029201

[B43] Pethick W. A. Stellingwerff T. Lacroix M. A. Bergstrom C. Meylan C. M. (2018). The effect of a team sport-specific heat acclimation protocol on plasma volume in elite female soccer players. *Sci. Med. Footb.* 2 16–22. 10.1080/24733938.2017.1384559

[B44] Ramanathan N. L. (1964). A New weighting system for mean surface temperature of the human body. *J. Appl. Physiol.* 19 531–533. 10.1152/jappl.1964.19.3.531 14173555

[B45] Rogers S. M. Baker M. A. (1997). Thermoregulation during exercise in women who are taking oral contraceptives. *Eur. J. Appl. Physiol. Occup. Physiol.* 75 34–38. 10.1007/s004210050123 9007455

[B46] Sawka M. N. Wenger C. B. Pandolf K. B. (1996). “Thermoregulatory responses to acute exercise–heat stress and heat acclimation,” in *Handbook of Physiology, Section 4: Environmental Physiology*, eds Fregly M. J. Blatteis C. M., (New York, NY: Oxford University Press), 157–185.

[B47] Senay L. C. (1979). Effects of exercise in the heat on body fluid distribution. *Med. Sci. Sports* 11 42–48. 481155

[B48] Senay L. C. Mitchell D. Wyndham C. H. (1976). Acclimatization in a hot, humid environment: body fluid adjustments. *J. Appl. Physiol.* 40 786–796. 10.1152/jappl.1976.40.5.786 931907

[B49] Stachenfeld N. S. (2008). Sex hormone effects on body fluid regulation. *Exerc. Sport Sci. Rev.* 36 152–159. 10.1097/JES.0b013e31817be928 18580296 PMC2849969

[B50] Stachenfeld N. S. Silva C. Keefe D. L. (2000). Estrogen modifies the temperature effects of progesterone. *J. Appl. Physiol.* 88 1643–1649. 10.1152/jappl.2000.88.5.1643 10797125

[B51] Stachenfeld N. S. Silva C. Keefe D. L. Kokoszka C. A. Nadel E. R. (1999). Effects of oral contraceptives on body fluid regulation. *J. Appl. Physiol.* 87 1016–1025. 10.1152/jappl.1999.87.3.1016 10484572

[B52] Stephenson L. A. Kolka M. A. (1985). Menstrual cycle phase and time of day alter reference signal controlling arm blood flow and sweating. *Am. J. Physiol.* 249(2 Pt 2), R186–R191. 4025576 10.1152/ajpregu.1985.249.2.R186

[B53] Sunderland C. Morris J. G. Nevill M. E. (2008). A heat acclimation protocol for team sports. *Br. J. Sports Med.* 42 327–333. 10.1136/bjsm.2007.034207 18460609

[B54] Sunderland C. Nevill M. (2003). Effect of the menstrual cycle on performance of intermittent, high-intensity shuttle running in a hot environment. *Eur. J. Appl. Physiol.* 88 345–352. 10.1007/s00421-002-0722-1 12527962

[B55] Taylor N. A. S. Cotter J. D. (2006). Heat adaptation: guidelines for the optimisation of human performance. *Int. Sport Med. J.* 7 33–57.

[B56] Tenaglia S. A. McLellan T. M. Klentrou P. P. (1999). Influence of menstrual cycle and oral contraceptives on tolerance to uncompensable heat stress. *Eur. J. Appl. Physiol. Occup. Physiol.* 80 76–83. 10.1007/s004210050561 10408316

[B57] Tounsi M. Jaafar H. Aloui A. Souissi N. (2017). Soccer-related performance in eumenorrheic Tunisian high-level soccer players: effects of menstrual cycle phase and moment of day. *J. Sports Med. Phys. Fitness* 58 497–502. 10.23736/S0022-4707.17.06958-4 28222573

[B58] Vescovi J. D. Favero T. G. (2014). Motion characteristics of women’s college soccer matches: Female Athletes in Motion (FAiM) study. *Int. J. Sports Physiol. Perform.* 9 405–414. 10.1123/IJSPP.2013-0526 24755966

[B59] Waldron M. Jeffries O. Tallent J. Patterson S. Nevola V. (2019). The time course of adaptations in thermoneutral maximal oxygen consumption following heat acclimation. *Eur. J. Appl. Physiol.* 119 2391–2399. 10.1007/s00421-019-04218-2 31512025 PMC6763528

[B60] Wiecek M. Szymura J. Maciejczyk M. Cempla J. Szygula Z. (2016). Effect of sex and menstrual cycle in women on starting speed, anaerobic endurance and muscle power. *Physiol. Int.* 103 127–132. 10.1556/036.103.2016.1.13 27030635

[B61] Willoughby D. S. Priest J. W. Nelson M. (2002). Expression of the stress proteins, Ubiquitin, heat shock protein 72, and Myofibrillar protein content after 12 weeks of leg cycling in persons with spinal cord injury. *Arch. Phys. Med. Rehabil.* 83 649–654. 10.1053/apmr.2002.31184 11994804

[B62] Wilson C. A. Abdenour T. E. Keye W. R. (1991). Menstrual disorders among intercollegiate athletes and non athletes: perceived impact on performance. *Athletic Train. JNATA* 26 170–177.

